# Going the extra mile at work: Relationships between working conditions and discretionary work effort

**DOI:** 10.1371/journal.pone.0288521

**Published:** 2023-08-02

**Authors:** Wei-hsin Yu, Janet Chen-Lan Kuo

**Affiliations:** 1 Department of Sociology, University of California, Los Angeles, California, United States of America; 2 Department of Sociology, National Taiwan University, Taipei City, Taiwan; St John’s University, UNITED STATES

## Abstract

Despite the implications of work effort for earnings inequality, rigorous and comprehensive analyses of how work conditions affect people’s tendency to exert extra work effort are rare. Using two waves of data from the National Longitudinal Survey of Youth 1997, this study examines how individuals’ discretionary work effort—i.e., effort in excess of what is required—changes with their work time, the tangible and intangible rewards from their jobs, and the social contexts of their occupations. Results from fixed-effects models show that frequently working in teams is associated with both women’s and men’s reported discretionary effort. Women also express a greater tendency to exert extra work effort when they work full time instead of part time and when their employers offer paid maternity leave, but less so when their occupations are male-dominant or require confrontations with people. Racial and ethnic minorities’ discretionary work effort changes in response to collaborative and competitive occupational environments somewhat differently from Whites. In addition, Black women’s tendency to exert excess work effort is less tied to their time spent on their jobs than White women’s. Beyond uncovering gender and ethnoracial differences, this study also underscores the need to consider the ways in which social aspects of work contribute to workers’ motivation and effort.

## Introduction

Social scientists have long been interested in the amount of effort individuals expend at work [1–8]. Those willing to put excess effort into work, much beyond the required level, are potentially more desirable in the labor market and, as a result, may receive higher pay. In this sense, individuals’ discretionary work effort, defined as effort in excess of what is required [3], has critical implications for earnings disparities. In fact, research on the gender pay gap often considers women’s and men’s different abilities and motivations to put extra effort at work, especially during their childrearing years, as one explanation for this gap [8–11].

Because discretionary work effort could potentially enhance job performance and earnings, it is important to understand what motivates or discourages people to “go the extra mile” at work. Many studies of work effort emphasize the roles of gender and family stages, with researchers debating on whether family responsibilities brought by marriage and parenthood increase men’s and decrease women’s discretionary work effort [1, 2, 4, 6–8, 12–14]. Beyond family roles, however, job conditions, which can shape workers’ expected rewards for their effort [15, 16], may also explain the engagement in discretionary effort. In Piore’s [17] classic writing about the “dual labor market,” for example, he suggests that people relegated to the secondary labor market, where jobs are insecure and lacking long-term prospects, learn not to value certain behavior, such as reliably showing up to work or exerting more work effort than required, because the secondary labor market does not reward such behavior. More recently, Kmec and Gorman [3] show that workers with more autonomous and secure jobs, which are likely in the primary labor market, report higher levels of discretionary work effort. Their finding echoes Piore’s argument that workers’ tendency to exert extra work effort is often responsive to structural characteristics of their jobs.

In spite of the potential importance of working conditions, few studies have comprehensively addressed which conditions are particularly relevant to individuals’ discretionary work effort or whether different groups of workers respond to the various conditions differently. In rare studies where researchers do investigate the roles of job and workplace characteristics [e.g., 3], they generally rely on cross-sectional comparisons of self-reported effort. Such comparisons cannot rule out the possibility that certain unobserved factors, such as people’s upbringings, value systems, and personality traits, simultaneously sort them to different types of jobs and determine their willingness to exert much effort at work. Moreover, people may have different reference groups and use differing standards to evaluate their own work effort, making the between-people comparisons of self-reported effort subject to measurement errors.

To shed light on the structural sources that motivate individuals’ hard work, or the lack thereof, in this study we use longitudinal data to examine how work conditions are linked to individuals’ tendency to put excess effort into work. To the best of our knowledge, all longitudinal studies about work effort use working hours to approximate such effort [8, 13]. Nevertheless, people may not have full control over the amount of hours they could work, and their hours spent at work may reflect their need for income more than their tendency to put extra effort into job tasks. In contrast, studies using self-assessed work effort tend to employ cross-sectional analyses [1, 3, 4, 18], which, as discussed above, face the potential problems of unobserved heterogeneity and inconsistent evaluation standards across individuals. We instead use observations of the same people at different time points to investigate how changes in their self-reported tendency to work extra hard correspond to alterations in their job experiences and environments. By doing so, we can better account for unobserved factors that both select individuals into different working conditions and affect their discretionary work effort or their self-assessments of such effort.

This study also exceeds prior research on work effort by more comprehensively considering different dimensions of paid work as sources for changes in discretionary work effort. Our analysis specifically focuses on how individuals’ work effort varies with their time spent on their jobs, the monetary and nonpecuniary rewards for their work, and the social contexts of their occupations. While nonmonetary benefits offered by employers and social environments surrounding workers can potentially affect individuals’ motivation to work hard, previous analyses of work effort generally do not address the roles of these factors [1, 3].

Because workers’ experiences may vary by their gender and race/ethnicity even at the same jobs, we further investigate how the link between paid work and work effort may differ between women and men and across ethnoracial groups. Unfavorable social dynamics at work may dampen women’s motivation to exert extra effort more than men’s, for example, given that research finds women to value social relations in the workplace more than men do [19, 20]. Likewise, certain working conditions such as frequently working in teams, which could compel individuals to work hard to earn and maintain their team members’ friendship [21], may matter more for Whites than for ethnoracial minorities, as the latter may feel less connected to their mostly White team members. As existing research on work effort predominantly focuses on gender differences and the effects of family responsibilities [1, 3, 4, 6, 12], our focus on labor market experiences and how the importance of these experiences differs across ethnoracial groups can further enrich the literature.

### Research on self-assessed and discretionary work effort

The literature on work effort unequivocally assumes that the amount of effort individuals devote to work can change over time. Human capital theory has long argued that family obligations, especially those associated with childrearing, constitute the main source of alterations in workers’ efforts [14], with mothers’ reduced work effort thought to explain their lower pay than childless women’s [9, 11]. Studies based on self-reported effort expended on the job, however, find that having a young child is associated with either a small decrease or no decrease in parents’ effort at work [2, 3], and that household responsibilities hardly reduce work effort, especially in the case of women [1, 12].

Job and workplace characteristics could also incentivize workers to exert more effort than their jobs’ requirements. Previous studies show that U.S. workers with higher-paying, more autonomous, and more secure jobs are more likely to expend extra effort at work [1, 3]. The commonly proposed rationale is that people are motivated to work harder when they receive more tangible or intangible rewards from their jobs. Nonetheless, because the research evidence on job rewards and self-reported work effort is derived from cross-sectional analyses, an alternative explanation is also plausible: that is, people with the personality to routinely exert maximum effort tend to land more rewarding jobs.

Not only does the cross-sectional nature of previous studies of self-reported work effort make the findings difficult to interpret, but their considerations of the dimensions of paid work that may affect discretionary work effort also rarely extend beyond the pecuniary rewards of jobs [2, 3]. Aside from providing compensations for workers’ labor, jobs also offer opportunities for social encounters. Much research suggests that social dynamics at work affect people’s job satisfaction and attachment [22–24]. Because more satisfied workers may be more willing and likely to expend extra effort on the job, the social context of work could also potentially shape individuals’ discretionary work effort.

### Working conditions and changes in discretionary work effort

Although longitudinal evidence about individuals’ tendency to exert excess effort at work is scant, existing theories on workers’ motivations and satisfaction suggest that time investment in paid work, pecuniary and nonpecuniary rewards from jobs, and the social context of work could all be relevant to workers’ choice between “going above and beyond” or simply meeting the minimum requirements at work [16, 22, 24–27]. We may therefore observe shifts in discretionary work effort as people experience variations in working conditions.

#### Work time and rewards

All jobs require time from workers and compensate for the time spent. There are, however, considerable variations in work time and rewards, which are likely to shape workers’ perceptions of their work effort and the worthiness of exerting effort. Starting from the number of hours at work, the gendered organization theory has long contended that employers’ belief in the ideal workers, who are willing to sacrifice time for all but their jobs, leads employers to equate working hours with effort [28, 29]. Because working hours are easily measurable, and because workers themselves may also be influenced by the prevailing image of ideal workers, they may think that they have higher standards and expend more effort than their peers at work when they work longer hours.

Alternatively, the number of work hours may be relevant to discretionary work effort because it has implications for workers’ economic prospects [30, 31]. The expectancy theory of motivation posits that individuals’ motivation to work hard depends on their belief in the extent to which their performance will result in desirable outcomes [15, 16]. Based on this theory, those whose job conditions signal a greater potential for wage increases and promotions should engage in discretionary work effort more. Because part-time jobs typically have worse long-term prospects than full-time jobs [32], those with part-time jobs may be less motivated to work harder than necessary. In contrast, jobs that require workers to spend far beyond the standard full-time hours—40 weekly hours—are thought to provide disproportionately high returns to workers’ time [30], which could elicit more discretionary work effort.

Many other job characteristics, such as high pay, elevated occupational prestige and advancement opportunities, and abundant job autonomy, could also signal a greater potential for job performance to be recognized and compensated, hence motivating workers to exert discretionary effort. Even if these desirable job characteristics are not reliable signals for future rewards, they, according to Goldthorp’s theory of class and employment relations [33], could still motivate workers’ discretionary effort as excess current compensations. Goldthorp argues that for jobs that are by nature difficult to monitor, such as managerial and professional positions, employers regulate employees with an implicit “service contract,” in which employers provide excess rewards in exchange for employees’ commitment and hard work. The idea that compensations beyond what the workers’ human capital mandates, in both pecuniary or nonpecuniary forms, can ensure productivity implies that excess rewards help cultivate intrinsic motivations in workers. As motivated workers are likely to put more effort than required, workers’ tendency to engage in discretionary effort can be expected to increase with their pay, occupational prestige and prospects, and autonomy at work.

Because fringe benefits are a crucial part of the compensation package for U.S. workers, how rewarded workers feel about their jobs can also depend on the benefits provided by their workplaces. More than wages or promotions, fringe benefits may be seen as an indicator of employers’ concern about employees’ welfare and not necessarily a prize for workers’ productivity, as benefits are typically offered to workers with a wide range of skills within organizations and can be extended to workers’ family members. Thus, fringe benefits may be relevant to workers’ discretionary effort not just because they signal future rewards or fulfill employers’ obligations in the service contract, but also because they convey “welfare corporatism,” in which employers generously cover employees’ needs outside of the workplace. Because welfare corporatism is conducive to workers’ commitment to their workplaces [34, 35], and committed workers tend to expend more effort on the job, we can expect that being entitled to abundant benefits will increase workers’ discretionary effort.

#### Occupational social contexts

Although the theories just discussed all focus on the rewards from jobs, psychologists have long argued that individuals’ intrinsic motivation and task effort depend on their relations with peers [36]. Most workers have to interact with people and be subjected to explicit and implicit comparisons with others at work. Social encounters and comparisons experienced by workers can determine how they feel about their jobs [22–24], thus shaping their discretionary effort. Much of people’s social experience at work has to do with their occupations’ specific tasks and arrangements. Some occupations, such as flight attendants and operation managers, nearly always work with a group or in a team, whereas others, such as postal mail carriers and craft artists, rarely involve teamwork. Similarly, some occupations require workers to compete intensely because the number of prizes in the profession is limited (e.g., realtors, who must compete for a fixed amount of properties or buyers), or because there are clear ways to compare workers’ performance (e.g., investment fund managers). Other occupations such as copy editors and physical therapists, by contrast, allow workers to achieve goals without directly competing with each other. As a result, the level of competitive pressure varies considerably across occupational contexts.

Psychological literature frequently debates on whether a collaborative or competitive work environment can better enhance individuals’ intrinsic motivation and work effort. While some studies find that cooperative work structures lead individuals to complete tasks more effectively [36], others show that a competitive environment cultivates industriousness more than a cooperative one for people who embrace individualistic values [37]. Yet another string of research demonstrates that imposing both collaborative and competitive goal structures in settings that previously used neither raises intrinsic motivations and performance [38, 39]. Although all these psychological studies are based on lab experiments, in real work settings where occupational contexts facilitate collaboration or competition, workers may similarly become more motivated and work harder. Following this logic, we can expect changes in workers’ discretionary work effort as they move to occupations that are under greater competitive pressure or emphasize teamwork more.

Beyond having a competitive or collaborative work atmosphere, different occupations also have implications for workers’ day-to-day encounters with others. Research shows that occupational gender composition is an important indicator of the social dynamics men and women experience in the workplace. Women in male-dominant occupations, in particular, often face skepticism and hostility from coworkers [40–42], making them less confident about their career choices [43]. In this context, women may find themselves disenchanted with their work lives and ultimately become unmotivated to exert excess effort at work. Moreover, occupations differ in their required activities, with some inevitably resulting in unpleasant social interactions. Police officers and bill collectors, for example, likely need to confront people and handle conflicts and aggression more often than library assistants and travel guides. The hostility and anger workers face in their occupations can potentially lower their satisfaction with work and hence discourage them from exerting discretionary work effort.

### Gender and ethnoracial differences

Although most research on gender disparities in work effort focuses on their potentially different responses to changes in family obligations, women’s and men’s effort could also be linked to working conditions in different ways. Prior research shows that men and women differ in how much they emphasize the extrinsic versus intrinsic rewards from work [20, 44]. Because men often focus on extrinsic rewards, such as pay, job autonomy, and advancement opportunities, it is likely that they will adjust their work effort according to alterations in job rewards. By contrast, women pay much more attention to intrinsic job rewards, which include positive social relations and interactions in the workplace [19]. A few studies further find that women’s personal-life attitudes and behavior are more sensitive to interpersonal interactions in the workplace than men’s [45, 46]. If women are indeed more concerned and more responsive to social dynamics at work, changes in occupational social contexts may also affect women’s discretionary work effort more than men’s.

Gender roles may also affect how individuals respond to their occupations’ gender compositions and confrontation requirements. Much research shows that women tend to suffer from hostile and unsupportive work environments when they are in male-dominant occupations, whereas men rarely report harassments or additional obstacles in female-dominant occupations [40–42, 47, 48]. This gender asymmetry suggests that women may experience discouragement and become unmotivated to put extra effort in customarily male occupations, but men would not face the same in customary female occupations. It is worth noting that Kmec and Gorman [3] find that both men and women in predominantly male jobs exert more discretionary effort than those in mixed-sex work settings. This finding, however, could be explained by fundamental personality differences between those obtaining male-dominated jobs and those obtaining other jobs, given that the researchers rely on cross-sectional data.

Because the gender system associates masculinity with aggression and femininity with friendliness and cooperativeness [49], women may feel less comfortable and encounter more resistance when they must confront angry and belligerent people in their occupations than men do. In turn, women may become disenchanted with work more easily in such occupations. Thus, occupational social contexts that involve conflicts and confrontations may be especially likely to be associated with lower work effort for women.

Beyond gender differences, we may find ethnoracial minority groups’ working conditions, especially those concerning the social environment, to be associated with their discretionary work effort differently from non-Hispanic Whites’. Because ethnoracial boundaries, as well as ethnoracial inequality, are reproduced and reinforced in everyday interactions [50], Whites and ethnoracial minority group may have differing experiences with their coworkers and perceive different career opportunities even in the same general social environment. Studies of managers, one occupation that frequently requires working with a group, find that minority managers receive less social support and have fewer close network ties [51, 52]. Other research shows that teamwork does not always benefit minority groups [53]. Racial and ethnic minorities may therefore feel less solidarity with coworkers and be less likely to exert extra effort even when they frequently work in a team. In addition, competitive work environments may discourage rather than encourage minorities to expend excess effort if they believe that their chance of winning is especially low. Previous research suggests minorities face more discrimination in more competitive work settings [54, 55], which could lower their motivation to work extra hard. We can therefore expect that racial and ethnic minorities to be less likely than Whites to exert more discretionary effort when they are in occupations that impose greater competitive pressure.

The relationship between working hours and discretionary effort may also differ for disadvantaged minorities because they have less control over their working hours. Blacks, in particular, are more likely than Whites to desire working more hours than they actually do [56, 57]. If the greater promise of future rewards is the reason why workers’ discretionary work effort may increase with their working hours, then all ethnoracial groups should report more effort as their working hours grow. However, if the link between working hours and work effort has to do with workers’ tendency to equate work time with effort, then we can expect those unable to work as many hours as they want to, such as Blacks, will not perceive themselves as less motivated or hard-working simply because they do not work long hours.

### Data

The data for the statistical analysis come from the National Longitudinal Survey of Youth 1997 (NLSY97), which collects information annually or biannually from a representative sample of U.S. men and women born in 1980 to 1984. We specifically selected the data from Rounds 12 and 14, conducted around 2009 and 2011, respectively, as these are the only rounds in which the NLSY97 asked a series of questions about respondents’ general tendency concerning work effort. The fact that the survey measures work effort twice enables us to observe its change over time. Because the two rounds of data were collected when respondents were in their mid-20s to early 30s, the vast majority of them had begun their careers. We can therefore examine how respondents’ work effort changes with shifts in their work conditions.

We selected the NLSY97 respondents who were interviewed in both Rounds 12 and 14 and had answered the questions about work effort in both rounds (79% of the entire sample). Although the survey collected information on the tendency regarding work effort from all respondents, we limit the analysis to those holding jobs in both rounds to make it easy to model and interpret the influences of jobs. Our earlier explorations indicated that changing from having a job to no job, or the other way around, did not affect individuals’ reported tendency regarding work effort. Incorporating those who hold no job in either round into the models also did not alter our main findings concerning the roles of work experiences and characteristics. We pooled the observations from both rounds to create a time-varying data set. After eliminating those without jobs in either round and those missing information on the key predictors, our analytic sample contains 10,654 person-round observations from 2,706 men and 2,621 women.

### Variables and measurement

For dependent variable used in the analysis, we constructed a composite measure of the tendency to engage in discretionary work effort from four items the NLSY97 included in both Rounds 12 and 14. Respondents specifically were asked the extent to which they agree with each the following statements: (1) I do not work as hard as the majority of people around me; (2) I do what is required, but rarely anything more; (3) I have high standards and work toward them; and (4) I make every effort to do more than what is expected of me. For each statement respondents chose on a 1–7 scale, with 1 being “disagree strongly” and 7 being “agree strongly.” After reverse coding items (1) and (2), we used principal component analysis to create an index to represent self-reported discretionary effort (eigenvalue = 2.05).

The key predictors for the analysis are all work-related. Starting from the time spent at work, we measured respondents’ hours spent on their current job per week, based their own reports. Following Cha and Weeden’s [30] study that distinguishes those working extremely long hours (i.e., “overwork”) from those whose working hours fall into the typical range of full-time jobs, we divided the weekly working hours into three categories: (1) part time (<35 hours), (2) standard full time (35–49 hours), and (3) overwork (> 50 hours). According to Cha and Weeden, those working 50 hours or longer tend to be perceived as especially committed and disproportionately rewarded. Such workers may internalize others’ perceptions and report a particularly great tendency to exert excess effort.

With regards to rewards from work, we included earnings, the level of recognition, the level of autonomy, and fringe benefits associated with respondents’ primary jobs. The NLSY97 asked respondents to report their yearly or weekly earnings and working hours and constructed the hourly pay based on the information. We converted back to total pay per week to include in our models because individuals’ sense of how well they are compensated is likely based on their total pay. To avoid the results being driven by the small number of respondents with extremely high earnings, we took the natural log of the weekly pay and used the transformed variable in the models. For the small proportion of person-years where the weekly earnings were missing (1.5%), we included a binary indicator for missing such information so that we can retain the cases in the analysis.

For the levels of recognition and autonomy, we derived the measures from the database compiled by the Occupation Information Network (O*NET), which was developed under the sponsorship of the U.S. Department of Labor. The O*NET database provides indicators of a wide range of occupational traits based on information collected from incumbents and experts for each occupation. By linking the NLSY97 respondents’ occupations with the O*NET occupations, we were able to measure various work characteristics that are unavailable in the NLSY97 data. In the analysis, we adopted the O*NET’s index for recognition—which summarizes the extent to which the occupation offers advancement, potential for leadership, and social prestige—with a minimum value of 1 and maximum value of 6.5. We constructed an index of job autonomy using four indicators from the O*NET: (1) the extent to which the job can be structured by the worker; (2) the frequency at which the worker makes decisions on the job; (3) the extent to which the worker has the freedom to make decisions at work; and (4) the extent to which the worker’s decisions can have impacts in the workplace. The O*NET reports all these indicators on a 1–5 scale. We standardized the distribution of each item and used the alpha scoring method to combine them into a single index of autonomy (Cronbach’s alpha = .87).

The NLSY97 asked respondents whether their jobs provide each of the following benefits: (1) childcare or a childcare subsidy, (2) a flextime work schedule, (3) paid maternal or parental leave, (4) unpaid maternal or parental leave, (5) medical insurance, (6) dental insurance, (7) life insurance, (8) a retirement plan, (9) stock options, and (10) tuition reimbursement for certain types of schooling. We coded a positive answer as 1, otherwise 0, for each item and used the alpha scoring method to combine the answers of the 10 items into an index of fringe benefits (Cronbach’s alpha = .88). In a further analysis, we also tested whether some types of fringe benefits are more effective in motivating workers than others. Nevertheless, because our earlier analysis indicated that medical insurance, dental insurance, life insurance, and a retirement plan are the most common and are frequently offered as a package, we could not examine their associations with reported discretionary effort separately. We therefore constructed an index of core benefits, which averages the binary indicators of these four types of benefits. We then examined how the provision of core benefits and each other type of benefit contributes to self-perceived work effort differently.

We introduced several variables related to the occupation’s social context, most of which are extracted from the O*NET database. First, to measure whether respondents’ occupational environments are relatively collaborative, we included the occupation’s level of emphasis on teamwork based on the O*NET’s rating, on a 1–5 scale, of how important it is for people on the job to work with a group or in a team. Second, we adopted the O*NET’s measure, also on a 1–5 scale, of the level of competitive pressure an occupation is subjected to. Third, to test whether an occupational context that involves conflicts and confrontations may weaken work effort, we created a composite measure from three indicators (each on a 1–5 scale) from the O*NET: (1) the frequency of dealing with unpleasant and angry people on the job, (2) the frequency at which those on the job are required to face physical aggression or violent people, and (3) the frequency of encountering conflict situations at work. We used the alpha scoring method to combine them into a single indicator of frequency of confrontations (Cronbach’s alpha = .81). Because the gender composition of the occupation can affect the interpersonal interactions men and women experience at work, we also measured the proportion of men in the occupation for the analysis. We derived this measure from the 2000 U.S. Census data and merged the information into the NLSY97 sample by linking the 3-digit occupational codes.

We included a series of time-varying controls that could shape attitudes about work, including work experience, education, family status, geographic location, and health conditions. Because workers at different career stages may have different levels of commitment, we measured respondents’ total length of work experience using the NLSY97’s record of the number of years each respondent spent on paid jobs since age 14. Education was measured in four categories based on the highest level respondents had completed at the time of interview: less than high school, high school, some college, and college and above. The models also took into account whether respondents were married, cohabiting, or unpartnered at the time of observation, as well as their number of children, as changes in family responsibilities can potentially alter individuals’ willingness to exert work effort [2, 14]. We further controlled for respondents’ region of residence (Northeast, Midwest, South, or West) and whether they lived in urban areas, based on the Census definitions for both. Finally, we measured respondents’ general health, which may affect how hard-working they can be. The NLSY97 respondents were asked to report about their general health from a 1–5 scale, with 1 being poor and 5 being excellent, and we used this report to indicate respondents’ health. To avoid dropping cases that have invalid values for any of the controls, we used a dummy variable to indicate missing values for educational level, relationship status, region, and urban residence, respectively. The missing values are less than 1% for most of the variables.

We also identified respondents’ gender and race/ethnicity to investigate gender and ethnoracial differences. The NLSY97 only provides two gender categories: women and men. For race/ethnicity, we classify respondents as non-Hispanic White, non-Hispanic Black, Hispanic, or other. We did not further distinguish those in the “other” category because of their relatively small number. For convenience we refer to non-Hispanic Whites as Whites and non-Hispanic Blacks as Blacks hereafter. Table 1 further provides descriptive statistics for all variables included in the analysis.

**Table 1:** Descriptive statistics of the analytic sample.

Variables	Mean/Percentage
Index of reported discretionary effort	0.09 (0.92)
Gender (%):	
Men	52.77
Women	47.23
Race/ethnicity (%):	
White	68.53
Black	13.46
Hispanic	12.99
Other	5.02
*Work time and rewards*	
Work hours (%):	
Part time	22.90
Standard full time	64.66
Overwork	12.44
Log weekly wage	6.15 (1.04)
Missing wage (%)	1.47
Recognition	3.29 (1.18)
Autonomy	0.30 (0.81)
Benefits	0.36 (0.31)
*Occupational social context*	
Teamwork requirement	4.19 (0.36)
Confrontation frequency	0.07 (0.95)
Proportion male	0.50 (0.29)
Competitive pressure	2.98 (0.52)
*Controls*	
Work experience (in years)	8.66 (2.74)
Education (%):	
Less than high school	6.66
High school	23.58
Some college	36.74
College and above	32.32
Unknown	0.70
Relationship status (%):	
Married	34.12
Cohabiting	20.73
Unpartnered	45.03
Unknown	0.12
Number of children	0.75 (1.07)
Region (%):	
Northeast	15.89
North Central	24.76
South	36.51
West	22.29
Unknown	0.55
Residence (%)	
Rural	21.03
Urban	75.68
Unclassified	3.30
Health	3.80 (.91)
N	10,654

Note: All numbers followed by parentheses are mean values, with standard deviations in parentheses. The remaining numbers are in percentage, as indicated in the table. The units of analysis are person-rounds.

### Analytic strategy

Although the objective of our study is to investigate how changes in work experiences and conditions contribute to alterations in one’s work effort, we began with a baseline model to show whether the self-assessed tendency to engage in discretionary work effort varies across gender and racial groups after accounting for differences in basic demographic characteristics. We fitted a population average model with a generalized estimating approach to estimate differences between gender and ethnoracial groups at the population level, while accounting for non-independence of observations from the same individuals.

Aside from the population average model, all our analysis used fixed-effects models, which can be expressed as:

$${WE}_{it}=\gamma_{0}+\Sigma a_{j}X_{jit}+\Sigma b_{k}Y_{kit}+\mu_{i}+\varepsilon_{it},$$

where the outcome is the tendency to exert discretionary effort for person *i* (*i* = 1, 2, 3, …, *n*) at year *t*; *γ*_0_ is the intercept; *X*_*jit*_ denotes *j* time-varying work-related variables and Σ*a*_*j*_ for their effects, *Y*_*kit*_ denotes *k* time-varying control variables, with Σ*b*_*j*_ indicating their effects; *μ*_*i*_ indicates fixed effects for *i* individuals in the sample; and *ε*_*it*_ is the error term. By including *μ*_*i*_, the models ultimately account for all differences between individuals that do not change over time, such as stable personality traits, early development of noncognitive skills, the family culture in which one grows up, and standards used to evaluate effort. Instead of showing how different people may have different tendencies to work harder than obligated by their jobs, our fixed-effects models demonstrate how changes in people’s work conditions correspond to alterations in their engagement in discretionary effort.

Because previous literature on work effort generally focused on whether factors associated with such effort differ between women and men [2, 3], we fit the fixed-effects models separately by gender. To examine ethnoracial differences, we further added interaction terms between work-related predictors and ethnoracial categories in the models. Although fixed-effects models do not allow us to include time-invariant variables such as race, it is possible to tell how time-varying factors may be associated with the outcome in different ways for various ethnoracial groups by including the interaction terms. To account for the NLSY97’s initial oversampling of minority groups and attrition over time, we applied the longitudinal weights generated for Rounds 12 and 14 in all models. We also estimated robust standard errors in conjunction with the use of weights.

## Results

Table 2 presents the baseline models in which only sociodemographic characteristics are included. The population average model shows that women reported a stronger tendency to exert discretionary work effort than men, but there is no ethnoracial difference after controlling for education, family conditions, geographical location, and health. Individuals with more education and in a more advanced career stage (as indicated by work experience) report a greater tendency to work harder than required. Married people also report a stronger tendency than the unpartnered. The fixed-effects models, however, indicate this result merely reflects unobserved differences between married and unpartnered people. Neither women nor men adjust their discretionary effort with changes in marital or parenthood status. The two groups are also similar in that they both report greater discretionary effort with increases in work experience. Thus, rather than those with a greater tendency to engage in discretionary effort participating in the labor market more than those without such a tendency, individuals indeed become more likely to go the extra mile at work as they accumulate more work experience.

**Table 2:** Baseline models predicting self-reported discretionary work effort.

	Population-average All	Fixed-effect Women	Fixed-effects Men	Gender Difference
Female	0.150*** (0.024)			
Race/Ethnicity (*ref*. White):				
Black	0.027 (0.032)			
Hispanic	0.028 (0.032)			
Other	0.038 (0.054)			
Work experience	0.033*** (0.004)	0.041*** (0.012)	0.052*** (0.011)	
Education (*ref*. < high school):				
High school	0.127* (0.056)	0.450* (0.177)	-0.151 (0.218)	*
Some college	0.168** (0.055)	0.487* (0.193)	-0.321 (0.265)	*
College and above	0.259*** (0.057)	0.611** (0.234)	0.025 (0.287)	
Unknown	0.356*** (0.101)	0.340 (0.225)	-0.229 (0.308)	
Relationship status *(ref*. married):				
Cohabiting	-0.039 (0.028)	0.033 (0.062)	-0.006 (0.065)	
Unpartnered	-0.125*** (0.025)	-0.016 (0.06)	-0.054 (0.062)	
Unknown	-0.097 (0.219)	0.054 (0.185)	-1.304* (0.618)	*
Number of children	0.018 (0.011)	-0.039 (0.044)	-0.022 (0.047)	
Region (*ref*. Northeast)				
North Central	-0.021 (0.039)	0.012 (0.141)	0.185 (0.167)	
South	0.055 (0.036)	0.151 (0.132)	0.312† (0.165)	
West	0.060 (0.038)	0.214 (0.159)	0.315† (0.171)	
Unknown	-0.246† (0.127)	0.249 (0.339)	0.249 (0.219)	
Residence (*ref*. rural)				
Urban	-0.047† (0.026)	0.045 (0.064)	-0.018 (0.055)	
Unclassified	-0.025 (0.049)	-0.094 (0.085)	0.074 (0.074)	
Health	0.113*** (0.011)	0.022 (0.022)	0.085*** (0.023)	*
Constant	-0.823*** (0.090)	-0.869*** (0.254)	-0.792** (0.286)	
N	10,654	5,242	5,412	

Note: Numbers in parentheses are robust standard errors. All models were estimated with the NLSY97 longitudinal weights for Rounds 12 and 14.

^†^ *p* < .10

* *p* < .05

** *p* < .01

*** *p* < .001

### Work conditions and women’s discretionary effort

To examine whether individuals’ tendency to exert excess work effort corresponds to changes in their work conditions, we added a series of work-related variables to the fixed-effects models. Table 3 shows the models for women. To show that the results for work-related factors are stable irrespective of model specifications, Models 1–3 contain somewhat different work-related predictors, with Model 4 including them all. The results for work conditions are consistent across the models. As Model 4 indicates, working full time, instead of part time, leads women to express a stronger tendency to exert discretionary effort. Overworking, however, is not associated with a significantly greater tendency than working standard full-time hours. Thus, only the switch between part-time and full-time work is meaningful to women’s self-assessed work effort. The salience of the divide between part-time and full-time perhaps reflects the lower expectations and opportunities workplaces typically have for part-time workers. The fact that devoting extremely long hours to their jobs does not raise women’s reported discretionary work effort suggests that women do not always equate working hours with self-motived effort. Thus, working hours may not be a good way to gauge female workers’ motivation and devotion.

**Table 3:** Fixed-effects models of women’s discretionary work effort on work-related conditions.

	Model 1	Model 2	Model 3	Model 4
*Work time and rewards*				
Work hours (*ref*. standard full time):				
Part-time	-0.108*			-0.106*
	(0.043)			(0.043)
Overwork	0.060			0.066
	(0.054)			(0.055)
Log weekly wage	-0.032*		-0.013	-0.029†
	(0.016)		(0.016)	(0.017)
Missing wage	-0.131		-0.146	-0.129
	(0.134)		(0.135)	(0.134)
Recognition	0.002		-0.005	-0.009
	(0.026)		(0.029)	(0.029)
Autonomy	-0.050		-0.033	-0.034
	(0.032)		(0.035)	(0.035)
Benefits	0.162**		0.198***	0.164**
	(0.060)		(0.060)	(0.060)
*Occupational social context*				
Teamwork requirement		0.164*	0.163*	0.160*
		(0.066)	(0.069)	(0.068)
Confrontation frequency		-0.077*	-0.069*	-0.071*
		(0.030)	(0.032)	(0.032)
Proportion male		-0.276*	-0.272*	-0.271*
		(0.124)	(0.125)	(0.123)
Competitive pressure		0.049	0.069	0.069
		(0.045)	(0.052)	(0.051)
*Controls*				
Work experience	0.042***	0.041***	0.040***	0.041***
	(0.012)	(0.012)	(0.012)	(0.012)
Education (*ref*. < high school):				
High school	0.419*	0.454*	0.413*	0.417*
	(0.183)	(0.18)	(0.18)	(0.186)
Some college	0.494*	0.492*	0.471*	0.489*
	(0.197)	(0.198)	(0.198)	(0.201)
College and above	0.591*	0.599*	0.575*	0.568*
	(0.240)	(0.241)	(0.241)	(0.244)
Unknown	0.354	0.278	0.254	0.286
	(0.226)	(0.235)	(0.235)	(0.233)
Relationship status *(ref*. married):				
Cohabiting	0.029	0.032	0.032	0.029
	(0.062)	(0.062)	(0.062)	(0.062)
Unpartnered	-0.012	-0.014	-0.007	-0.010
	(0.060)	(0.060)	(0.060)	(0.060)
Unknown	0.130	0.089	0.098	0.150
	(0.196)	(0.199)	(0.231)	(0.202)
Number of children	-0.026	-0.038	-0.031	-0.026
	(0.043)	(0.044)	(0.043)	(0.043)
Health	0.021	0.024	0.025	0.024
	(0.022)	(0.022)	(0.022)	(0.022)
Constant	-0.694*	-1.608***	-1.612***	-1.455***
	(0.284)	(0.391)	(0.405)	(0.404)
N	5,242	5,242	5,242	5,242

Note: Numbers in parentheses are robust standard errors. The NLSY97 longitudinal weights for Rounds 12 and 14 are used to estimate the models. The models also control for region and urban (versus rural) residence.

^†^ *p* < .10

* *p* < .05

** *p* < .01

*** *p* < .001

With respect to job rewards, surprisingly few types of rewards are important. Contrary to the expectancy theory or service contract argument, which sees ample rewards as a way to inspire employees’ excess effort, moving to jobs that pay better, allow greater recognition and prospects, or provide more autonomy does not alter women’s tendency to engage in discretionary effort. Working for firms with more extensive fringe benefits, however, is positively associated with this tendency. This finding is consistent with the welfare corporatism perspective that when employers are seen as benevolent and caring, they are more likely to gain employees’ commitment and discretionary work effort.

To understand which type of fringe benefits is more relevant to discretionary work effort, we further fit fixed-effects models in which we introduce different types of fringe benefits one by one. Table 4 presents the results from the models, but we omit the coefficients for all the other variables (which are same as in Model 4 in Table 2) to conserve space. The results show that few benefits take away the positive association between the core benefits, which includes medical and dental insurance and a retirement plan, and women’s reported work effort. The only exception is paid maternity or parental leave. After adding the provisions of paid and unpaid parental leave in Model 4, the coefficient of the core benefits weakens, to the extent that it is no longer significantly associated with women’s reported work effort. Meanwhile, switching to jobs that provide paid maternal or parental leave is clearly linked to increased discretionary effort for women in our sample, who were in an age range when childbirths are common. Thus, of all the welfare policies in the workplace, providing paid parental leave appears to be the most effective at eliciting discretionary effort from women of childbearing age.

**Table 4:** Fixed-effects models of women’s discretionary work effort on job benefits.

	Model 1	Model 2	Model 3	Model 4	Model 5	Model 6	Model 7
Core benefits	0.102*	0.097*	0.100*	0.035	0.081†	0.117*	0.117*
	(0.045)	(0.046)	(0.046)	(0.053)	(0.047)	(0.049)	(0.049)
Childcare		0.031					
		(0.053)					
Flextime			0.006				
			(0.034)				
Paid parental leave				0.089*			
				(0.037)			
Unpaid parental leave				0.051	0.082		
				(0.036)	(0.062)		
Stock option						-0.036	
						(0.041)	
Tuition subsidy							-0.036
							(0.041)

Note: Numbers in parentheses are robust standard errors. All models contain the other predictors in Model 4 of Table 2, including work experience, work hours, wages, recognition, autonomy, teamwork requirement, frequency of confrontation, proportion male, competitive pressure, relationship status, number of children, region, urban residence, and health, but we omit their coefficients to conserve space. The total number of person-years in the models is 5,242. The NLSY97 longitudinal weights for Rounds 12 and 14 are applied to each model.

^†^ *p* < .10

* *p* < .05

** *p* < .01

*** *p* < .001

Turning back to Table 3, several results concerning occupational social contexts are notable. To begin, working in occupations that emphasize teamwork is associated with a stronger tendency to exert excess effort for women. This finding is consistent with the argument that individuals are likely to become more industrious in collaborative work environments, where meeting their teammates’ expectations serves as a motivation to work harder. Model 4 also shows that women’s reported discretionary effort decreases when their occupations require more frequent confrontations with others. Similarly, women’s self-perceived work effort declines as they move to occupations with a higher proportion of men. It is possible that both daily confrontations and incongruency between women’s gender and their occupation’s gender image make work environments less pleasant for women, which discourages them from working harder than obligated.

### Work conditions and men’s discretionary effort

Table 5 presents a series of fixed-effects models examining how various work experiences and conditions are associated with men’s tendency to exert excess effort. Similar to women, men’s reported tendency to put in extra effort strengthens with increases in their occupations’ emphasis on teamwork. Hence, a collaborative environment is also conducive to an increase in the determination to expend discretionary effort for men. Changes in other work-related factors are not significantly associated with shifts in men’s reported work effort as they do women’s, although the magnitudes of the gender differences in the coefficients do not always reach the statistical significance level. Men and women appear to differ in how responsive their work effort is to the workplace benefits they are entitled to. As revealed earlier, the provision of paid parental leave is the main benefit conducive to the tendency to work extra hard for women. It is understandable that the same benefit does not affect men, given that men rarely take advantage of parental leave even when it is available [58].

**Table 5:** Fixed-effects models of men’s discretionary work effort on work-related conditions.

	Model 1	Model 2	Model 3	Model 4	Gender differences#
*Work time and rewards*					
Work hours (*ref*. standard full time):					
Part-time	-0.017			-0.013	
	(0.047)			(0.047)	
Overwork	0.087†			0.093†	
	(0.051)			(0.05)	
Log weekly wage	0.005		0.010	0.005	
	(0.020)		(0.020)	(0.020)	
Missing wage	-0.072		-0.105	-0.090	
	(0.164)		(0.166)	(0.164)	
Recognition	0.017		0.016	0.008	
	(0.026)		(0.028)	(0.028)	
Autonomy	0.028		0.039	0.032	
	(0.034)		(0.039)	(0.038)	
Benefits	0.013		0.018	0.003	†
	(0.071)		(0.070)	(0.071)	
*Occupational social context*					
Teamwork requirement		0.178**	0.159*	0.165**	
		(0.06)	(0.063)	(0.063)	
Confrontation frequency		-0.002	-0.017	-0.015	
		(0.029)	(0.031)	(0.031)	
Proportion male		-0.010	-0.045	-0.037	
		(0.101)	(0.104)	(0.102)	
Competitive pressure		-0.004	-0.041	-0.039	
		(0.050)	(0.061)	(0.061)	
*Controls*					
Work experience	0.050***	0.052***	-0.074	0.050***	
	(0.011)	(0.011)	(0.220)	(0.011)	
Education (*ref*. < high school):	-0.154	-0.130	-0.181	-0.137	
	(0.219)	(0.219)	(0.263)	(0.220)	
High school	-0.313	-0.301	0.207	-0.299	
	(0.265)	(0.264)	(0.283)	(0.264)	
Some college	0.013	0.033	-0.056	0.022	*
	(0.286)	(0.285)	(0.304)	(0.285)	
College and above	-0.220	-0.220		-0.215	
	(0.306)	(0.305)		(0.304)	
Unknown					
Relationship status *(ref*. married):					
Cohabiting	-0.002	-0.009	-0.047	-0.006	
	(0.065)	(0.065)	(0.065)	(0.065)	
Unpartnered	-0.044	-0.056	-0.098	-0.048	
	(0.062)	(0.062)	(0.061)	(0.062)	
Unknown	-1.309*	-1.317*	-1.320*	-1.322*	*
	(0.618)	(0.614)	(0.58)	(0.614)	
Number of children	-0.016	-0.020	0.051	-0.016	
	(0.046)	(0.047)	(0.044)	(0.047)	
Health	0.084***	0.086***	0.078***	0.085***	*
	(0.023)	(0.023)	(0.023)	(0.023)	
Constant	-0.869**	-1.504***	-1.032*	-1.383**	
	(0.308)	(0.405)	(0.435)	(0.444)	
N	5,412	5,412	5,412	5,412	

Note: Numbers in parentheses are robust standard errors. The NLSY97 longitudinal weights for Rounds 12 and 14 are used to estimate the models. The models also control for region and urban (versus rural) residence.

# indicates t-test results between coefficients in Model 4 with those in Model 4 in Table 2.

^†^ *p* < .10

* *p* < .05

** *p* < .01

*** *p* < .001

### Ethnoracial differences

While the results discussed thus far demonstrate important gender differences, the fixed-effects models with the entire male or female sample could mask ethnoracial differences. We therefore fitted additional models with interaction terms between all work-related variables and ethnoracial categories. We then retained only the interaction terms that generated statically meaningful results in the final models. Table 6 presents these final models for women and men, with the coefficients for all control variables omitted to conserve space.

**Table 6:** Fixed-effects models predicting work effort, focusing on ethnoracial differences.

	Women	Men
*Work time and rewards*		
Work hours (*ref*. standard full time):		
Part time	-0.167**	-0.014
	(0.054)	(0.047)
Overwork	0.085	0.095†
	(0.066)	(0.051)
Part time × Black	0.366***	
	(0.091)	
Part time × Hispanic	0.082	
	(0.109)	
Part time × other race	-0.136	
	(0.175)	
Overwork × Black	-0.062	
	(0.162)	
Overwork × Hispanic	-0.084	
	(0.126)	
Overwork × other race	-0.069	
	(0.269)	
Log weekly wage	-0.030†	0.004
	(0.017)	(0.02)
Missing wage	-0.127	-0.098
	(0.133)	(0.164)
Recognition	-0.011	0.008
	(0.029)	(0.028)
Autonomy	-0.053	0.026
	(0.043)	(0.039)
Autonomy × Black	0.146*	
	(0.069)	
Autonomy × Hispanic	0.049	
	(0.067)	
Autonomy × other race	-0.165	
	(0.137)	
Benefits	0.163**	0.011
	(0.061)	(0.071)
*Occupational social context*		
Teamwork requirement	0.161*	0.238**
	(0.068)	(0.076)
Teamwork × Black		-0.248
		(0.166)
Teamwork × Hispanic		-0.031
		(0.154)
Teamwork × other race		-0.524*
		(0.220)
Confrontation frequency	-0.069*	-0.015
	(0.031)	(0.031)
Proportion male	-0.248*	-0.025
	(0.122)	(0.102)
Competitive pressure	0.134*	-0.042
	(0.066)	(0.061)
Competitive pressure × Black	-0.264**	
	(0.099)	
Competitive pressure × Hispanic	-0.160†	
	(0.093)	
Competitive pressure × other race	-0.262†	
	(0.157)	
N	5,242	5,412

Note: Numbers in parentheses are robust standard errors. All models control for socioeconomic factors including relationship status, number of children, region, urban residence, and health. We omit the coefficients for these factors to conserve space. The NLSY97 longitudinal weights for Rounds 12 and 14 are applied to each model.

^†^ *p* < .10

* *p* < .05

** *p* < .01

*** *p* < .001

The model for women indicates that whereas working full time, as opposed to part time, leads White women to report more discretionary work effort, it does not do the same for Black women. In fact, Black women working part time expressed a greater tendency to engage in discretionary effort than their counterparts with full-time jobs (*p* < 0.05). This finding is congruent with the idea that disadvantaged groups’ frequent inability to obtain jobs that offer as many working hours as they want makes them likely disassociate working hours from the amount of effort they tend to exert at work. Although our earlier models showed women’s self-assessed work effort does not correspond to their occupation’s level of competitive pressure (Table 2), the model here demonstrates that competitive pressure is actually relevant to Whites’ reported effort. Whereas being in a competitive occupational context enhances White women’s determination to exert effort beyond the required minimum, it does not motivate minority women to do the same. Thus, for White women, our findings are consistent with the psychological literature that argues both cooperative and competitive environments motivate workers to exert more effort. However, this pattern does not apply to other ethnoracial groups. All non-White women’s effort appears to be negatively affected when their work environments become more competitive. This result is consistent with the argument that minority groups feel especially disadvantaged in competitive work contexts; therefore, they may be less willing to expend effort to compete for the rare prize.

Table 6 further shows that the only interactions that yielded somewhat significant results for men are the ones with the occupation’s emphasis on teamwork. The positive association between the teamwork requirement and work effort might especially apply to White men, who are significantly different from Non-Black non-Hispanic minority men in this regard. In fact, when we calculated the total effect of occupational emphasis on teamwork for Black, Hispanic, and other minority men using the coefficients from the model presented in Table 5, we found this emphasis has no statistically significant effect on all these minority groups’ work effort. Taken together, these results suggest that White men, who are more likely to receive support from and feel connected to coworkers than men in minority groups, are particularly likely to be motivated to exert extra effort in collaborative environments.

## Conclusions

Because individuals’ tendency to exert discretionary effort at work affects their productivity and earnings, understanding factors contributing to variation in this tendency is important for our knowledge of social inequality. As far as we are aware, this study is the first to use longitudinal data to examine people’s tendencies to expend effort and meet standards beyond their jobs’ requirements. We have shown that individuals’ reported tendency to work harder than required varies with some of their job conditions, with women’s discretionary work effort appearing more sensitive to alterations in work characteristics. Specifically, frequently working in teams increases reported discretionary effort for both women and men. Women also report to put more effort when they work full time instead of part time and when they work for employers who offer paid maternity leave. In addition, working in occupations that require frequent confrontations with people and have a disproportionately large share of men are significantly tied to a reduced tendency to exert excess work effort for women, but not for men.

By employing a longitudinal analysis, our study provides more rigorous evidence about how women and men modify their work effort in response to changes in their work and personal lives than prior research does. Several of our results clearly contradict those derived from previous cross-sectional analyses. Whereas an earlier study indicates that both men and women in male-dominant occupations exert more discretionary work effort than in other occupations [3], we show that the share of men in the occupation is relevant to only women’s work effort and that women’s tendency to apply extra effort actually lessens with an increase in men’s share in their occupation. Likewise, prior research suggests that providing greater job autonomy and offering higher pay inspire workers to expend more effort [1, 3], but we find that such rewards do not motivate individuals to work harder than obligated. The only job reward that is meaningful enough to elicit excess work effort from women, at least women of childbearing age, is the provision of paid parental leave. It is likely that the findings from earlier studies reflect the sorting of people who possess an especially firm belief in high work effort—say, those of type A personality—into male-dominant, high-paying, and relatively autonomous jobs. Only when we account for this sorting with fixed-effects models can we know how people’s discretionary work effort varies with working conditions.

Beyond providing better evidence, our findings that women’s reported work effort is responsive to the fringe benefits offered to them, but not other rewards such as pay, advancement opportunities, and autonomy have important theoretical implications. To be specific, the lack of importance of pay, recognition and advancement opportunities, and job autonomy directly contradicts the expectations derived from the expectancy theory or service contract argument. Instead, our findings offer support for the welfare corporatism perspective and highlight the need to distinguish among various types of job rewards. The main difference between fringe benefits and compensations including pay, recognition, and autonomy is that the latter group of rewards are more tied to workers’ qualifications. Workers are therefore likely to feel entitled to the pay, recognition, and autonomy that come with their jobs. Conversely, fringe benefits reflect employers’ attempt to take care of employees irrespective of their relative contributions to the workplace; fringe benefits thus enhance employees’, at least women employees’, motivations and work effort more than other types of rewards.

Despite the differing results from prior research regarding which work characteristics matter, our finding that women’s work effort fluctuates with their labor market experiences but not with changes in family roles is congruent with various sociological studies’ [2, 3, 12]. With longitudinal data, we are able to provide stronger evidence that rises in family responsibilities with marriage and childbearing do not weaken women’s determination to exert effort at work. Contrary to human capital theorists’ claim [14], gendered family roles are not the root of women’s adjustments in work effort. Nevertheless, our finding that women’s reported work effort varies more with changes in their work conditions does suggest that women adjust their work effort more frequently than men. It is likely that women reflect more often than men on whether their jobs are worthy of excess effort because they value jobs’ intrinsic rewards, including the meaningfulness and social pleasantness, more than men do [19, 20].

This study also contributes to the literature on work effort by uncovering crucial ethnoracial differences. We show that the hours spent at work are not linked to Black women’s perceived discretionary effort as they are for Whites. This finding, which we suggest has to do with disadvantaged minority women’s difficulty obtaining as much work as they are willing to do, has a particularly important implication for our understanding of work effort. Specifically, the loose connection between self-evaluated discretionary work effort and working hours implies that the common practice of treating working hours as a proxy for work effort in existing literature is likely to yield especially inaccurate conclusions about disadvantaged minority women [e.g.,^7^, ^8^].

In addition, our analysis demonstrates that working in competitive occupational contexts motivates White women, but not minority women, to exert more work effort. Being in occupations that emphasize teamwork more appears to elicit extra effort from men only if they are White. Taken together, these results suggest that the arguments about how peer relations in the work environments are conducive to the development of industriousness are generally more relevant to Whites than minorities. Future research needs to pay more attention to how Whites and minorities could have different personal experiences that affect their work-related values and efforts even within similar occupational contexts.

Although our longitudinal analysis accounts for much unobserved heterogeneity, we must acknowledge that fixed-effects models do not help us ascertain the causal order. We cannot completely rule out that instead of jobs of given characteristics shaping individuals’ work effort, it is a change in the tendency to put extra effort that enables individuals to obtain certain types of jobs. Several of our results, however, suggest that the reverse causality is unlikely. For example, it is difficult to explain why a lower tendency to exert excess effort would facilitate women’s move to occupations with higher proportions of men, given that women often have to work harder than their peers to prove themselves in male-dominant occupations [42, 43]. Similarly, it is hard to interpret our finding about the Black–White difference in the relationship between work effort and occupation’s competitive pressure if work effort is the cause; it seems unlikely that White women would need to exert more discretionary effort to enter more competitive occupations, but Black women would not.

Another limitation we face is that self-reported discretionary work effort can be subjective and vary by respondents’ reference groups or their learned ability to accurately assess their own effort. Even with the fixed-effects modeling approach, we must acknowledge that individuals’ reference groups and accuracy in reporting their own effort could change over time. Such unobserved time-varying factors could be related to both individuals’ work conditions and self-assessed discretionary effort, thus affecting the interpretations of our results. The fact that the two waves of data used in the study were collected only two years apart, however, should help alleviate this concern, as individuals’ views about what makes one a hard-working person and what constitutes a high performance standard are unlikely to change drastically during a short period of time. As for potential changes in reference groups, we conducted an additional analysis in which we removed the item that asks respondents to explicitly compare themselves to others (“I do not work as hard as the majority of people around me”) from the measure of discretionary work effort, and the results remained similar. This additional analysis adds to our confidence that our main results do not just reflect individuals changing reference groups as their move to different jobs.

At a more general level, this study contributes to our knowledge of work by paying special attention to different social contexts across occupations. We find that workers, especially women, alter their discretionary effort according to the social dynamics they experience at work, suggesting that interpersonal relations and interactions in the workplace serve as important enhancers or depressors of workers’ effort. In this sense, remote working from home, which has become prevalent since the onset of the COVID-19 pandemic, could have critical effects on workers’ motivations and productivity, as it makes the social interactions and comparisons that typically occur in the workplace seem more distant and abstract. Thus, future research on the consequences of home-based work arrangements, as well as the influences of jobs on individuals in general, should focus more on the social aspects of work as well as how changes in these aspects could affect individuals’ values and behavior.
